# Community health insurance schemes & patient satisfaction - evidence from India

**Published:** 2011-01

**Authors:** N. Devadasan, Bart Criel, Wim Van Damme, Pierre Lefevre, S. Manoharan, Patrick Van der Stuyft

**Affiliations:** * Institute of Public Health, Bangalore & Achutha Menon Centre for Health Science Studies, SCTIMST, Thiruvananthapuram; ** Department of Public Health, Institute of Tropical Medicine, Antwerp, Belgium; + ACCORD, Gudalur, India

**Keywords:** Community health insurance, India, micro health insurance, patient satisfaction, quality of care

## Abstract

**Background & objectives:**

Quality of care is an important determinant for utilizing health services. In India, the quality of care in most health services is poor. The government recognizes this and has been working on both supply and demand aspects. In particular, it is promoting community health insurance (CHI) schemes, so that patients can access quality services. This observational study was undertaken to measure the level of satisfaction among insured and uninsured patients in two CHI schemes in India.

**Methods:**

Patient satisfaction was measured, which is an outcome of good quality care. Two CHI schemes, Action for Community Organisation, Rehabilitation and Development (ACCORD) and Kadamalai Kalanjiam Vattara Sangam (KKVS), were chosen. Randomly selected, insured and uninsured households were interviewed. The household where a patient was admitted to a hospital was interviewed in depth about the health seeking behaviour, the cost of treatment and the satisfaction levels.

**Results:**

It was found that at both ACCORD and KKVS, there was no significant difference in the levels of satisfaction between the insured and uninsured patients. The main reasons for satisfaction were the availability of doctors and medicines and the recovery by the patient.

**Interpretation & conclusions:**

Our study showed that insured hospitalized patients did not have significantly higher levels of satisfaction compared to uninsured hospitalized patients. If CHI schemes want to improve the quality of care for their clients, so that they adhere to the scheme, the scheme managers need to negotiate actively for better quality of care with empanelled providers.

Quality of care is one of many important determinants of health service utilization. Various studies show that health services’ utilization is sensitive to the perception of quality by the users^1^–^4^. While many articles concentrate on the technical aspects^5^–^8^, studies are increasingly looking at quality from the patient’s perspective^1^,^4^,^9^.

The quality of healthcare in India in both the private and public health sector is unsatisfactory^10^. The problems include non-availability of staff and medicines as well as the rude behaviour of the staff^5^. Studies in the private sector have shown that practitioners tend to prescribe unnecessary and even harmful medicines^8^,^11^. Recent policy documents also acknowledge the lack of quality in the Indian health services^12^,^13^. One of the recommended strategies is to introduce demand-side financing, specifically community health insurance (CHI)^13^.

There are three possible mechanisms whereby CHI can improve the quality of care. One of the mechanisms is when the organizer of the CHI scheme strategically purchases health care from the provider^14^. Strategic purchasing includes among other facets, a mandate to set quality standards of care. This could include the following activities: gate keeping, contracting out with specific providers, maintaining a provider profile and monitoring the quality and financial performance, conducting utilization reviews, quality assurance, introducing generic medicines and implementing standard treatment protocols^15^. To summarise, the organizer of the scheme can negotiate with the provider for ‘better quality of care’ because they control the funds and are ultimately responsible for paying the provider. Yet another mechanism is by empowering the community. In any health insurance scheme, there is an element of ‘service guarantee’ *i.e*. once the insured pays the premium, the insurer has to guarantee the promised services. This can then give the insured patient the authority to ‘demand’ the services from the provider. Thus, ideally the insured patient can access the care that is required. A third mechanism is from the provider side. Especially in the Indian milieu where the private practitioners compete with each other for patients, providers would be happy to empanel themselves with a CHI scheme and have a captive community of patients who would use their services. This would ensure that they receive a steady income over time. They would thus be willing to improve their standard of care, to ensure that they remain empanelled with the CHI scheme. Thus insured patients should hypothetically receive better quality of care from these providers.

However, there is very little evidence that this relationship between CHI schemes and improved quality of care actually exists^16^,^17^. Ranson showed that some insured women at self Employed Women’s Association (SEWA) were exposed to ‘dangerous’ hospital conditions while undergoing hysterectomy^18^. A study in China also documented that insured patients under the New Comprehensive Medical Scheme were exposed to over prescribing compared to uninsured patients^19^. This suggests that community health insurance could potentially lead to patients using facilities that provide poor quality care.

This study is part of a larger study on the performance of Indian CHI schemes. The effect of CHI was assessed on quality of care using patient satisfaction as a proxy. Patient satisfaction is an important but little studied aspect of quality of care in the Indian context. Satisfaction is defined as the “overall level of contentment with a service experience”^20^. Two CHI schemes were studied between 2004 and 2005, one with a single provider and the other with multiple empanelled providers. The objective was to see whether insured patients have higher satisfaction levels as compared to the uninsured patients. Also the reasons for this satisfaction / dissatisfaction were explored. The underlying hypothesis was that insured patients would be more satisfied as they receive ‘better quality of care’.

## Material & Methods

*Action for Community Organisation, Rehabilitation and Development (ACCORD) - Association for Health Welfare in the Nilgiris (ASHWINI) - Adivasi Munnetra Sangam (AMS) CHI scheme:* ACCORD, a non-governmental organization (NGO) in Tamil Nadu, south India, works for the overall development of the indigenous people of Gudalur sub-district. This population also called ‘adivasis’ has traditionally been a hunter - gatherer society. As per the 2001 Census, there were 14,149 adivasis in Gudalur^25^. ACCORD collaborates with a community-based organization, the Adivasi Munnetra Sangam (AMS), to fight for adivasi rights. In addition, ACCORD provides health, education and agricultural services for the adivasis.

ACCORD’s health programme (ASHWINI) is a three-tier health system, with village health workers, health centres and a 20-bed hospital. Other than the ASHWINI hospital, there are four NGO hospitals with a total of 75 beds - three government hospitals (160 beds) and one private hospital (10 beds) in Gudalur sub district.

**Fig. 1:**
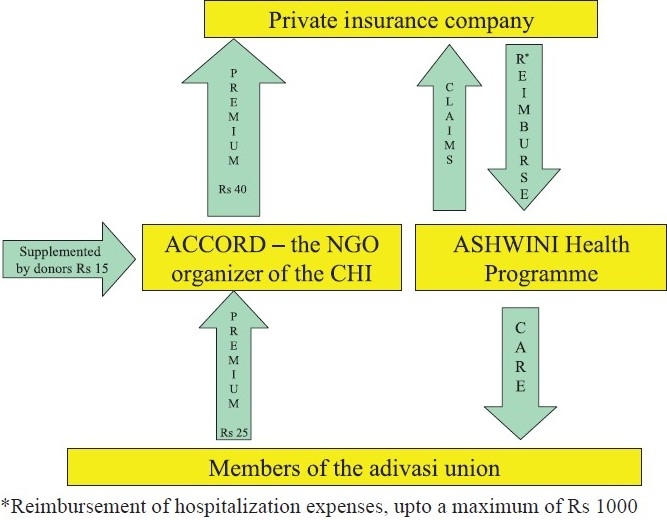
The ACCORD-ASHWINI-AMS community health insurance scheme in 2004. ACCORD, Actions for Community Organisation, Rehabilitation and Development; ASHWINI, Association for Health Welfare in the Nilgiris; AMS, Adivasi Munnetra Sangam.

Part of the ACCORD health service was financed by a CHI scheme initiated in 1992. All AMS members and their households are eligible to join the ACCORD CHI scheme (Fig. 1). In 2004, each AMS member paid a premium of 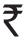 25 (US$0.57) per person per year during a definite annual collection period. This premium was collected by ACCORD and ASHWINI field staff and AMS leaders. Primary care was provided free to all adivasis, irrespective of their insurance status, by health staff at village and health centre levels. Insured members, if hospitalized in the ASHWINI hospital (after a waiting period of one month), were entitled to hospital care up to a maximum limit of 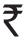 1,000 (US$ 23). Insured members hospitalized elsewhere did not receive any reimbursement of costs. Uninsured AMS members had to pay the cost of medicines when treated at the ASHWINI hospital.

*The Kadamalai Kalanjiam Vattara Sangam (KKVS) CHI scheme:* Development for Humane Action (DHAN) is a professional development organization started in 1997. Its main objective is to bring motivated youth to the development sector. DHAN manages various programmes, the main one being community banking through women-led self-help groups. These village-level groups are federated into ‘Kalanjiams’ at the sub-district level. In 2004, there were 46 Kalanjiams providing credit to 262,903 women in 5,054 villages.

One such federation is the Kadamalai Kalanjiam Vattara Sangam (KKVS). In 2004, it was a federation of 5391 women members spread over 65 villages in the Kadamalai sub-district of Theni district, Tamil Nadu. The KKVS was unique among all the federations as it had piloted a CHI scheme for its members. All women members and their families (between the age of 0 and 55 yr) were eligible to enrol for this CHI scheme. To enrol, they had to pay an annual subscription fee of 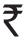 100 (US$ 2.3) per individual or 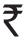 150 (US$ 3.2) per family. This fee was collected by the women’s groups every April and handed over to the KKVS insurance committee.

**Fig. 2:**
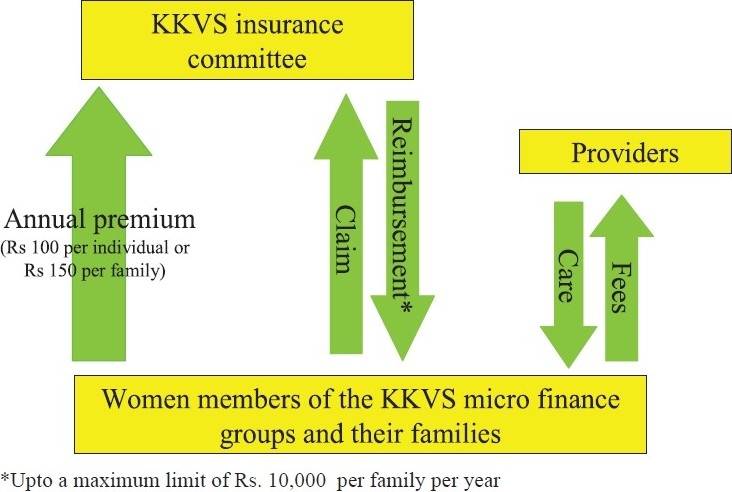
The KKVS community health scheme in 2005. KKVS, Kadamalai Kalanjiam Vattara Sangam.

Enrolled individuals could, after a waiting period of one month, access hospital care in any of the eight empanelled hospitals at Kadamalai or Theni, provided that they were referred by the KKVS primary centre. The patient was expected to pay the bills and submit relevant documents to the insurance committee (Fig. 2). After scrutiny and if found valid, 75 per cent of the claim was reimbursed, up to a maximum of 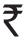 10,000 (US$ 228).

*Patient satisfaction framework:* Campbell has constructed a framework to demonstrate the links between various elements of quality of care^21^. In this framework, patient satisfaction is shown as an outcome of good quality care. Satisfaction is determined by service quality, customer expectations, subjective disconfirmation and emotions experienced during service delivery. Thus patient satisfaction gives an important insight into the quality of care provided by the health services.

There are many frameworks to assess patient satisfaction^22^. The only one that has been tested in a low income country was the framework developed and validated in Bangladesh^20^. The authors used six variables with various measures for each variable. Using this framework as a basis, we identified the measures through a mixture of literature review and focus group discussions (FGD) with the local stakeholders. Some of the indicators, *e.g*. “warmly received, waiting time, examination, *etc*.” were mentioned in the literature^1^,^4^,^23^. Based on these findings, a comprehensive list of 19 measures was developed (Table I). Each indicator was measured using a dichotomous scale through a structured questionnaire. Other than the questions on the afore-mentioned indicators, patients were also asked openended questions as to why they were (or were not) satisfied with the care received.

**Table I:** Framework of indicators for patient satisfaction

Variable	Measure
Overall satisfaction	The medicines were effective.
	I was satisfied with the care.
	I feel better now.
	I felt cared for.
Doctor’s service orientation	Had faith in the doctor.
	The doctor listened to my problems.
	The doctor examined me.
	The doctor explained to me about my
	condition.
	I received discharge instructions.
Nurse’s service orientation	I was received warmly.
	I was not shouted at.
	I was not afraid.
	The staff was courteous.
Tangibles (hospital and staff) Processes	Received medicines.
	Amenities were available.
	The waiting time was not long.
	Visitors were allowed to see me.
	Did not have to pay tips.
	Treatment was not costly.

**Fig. 3:**
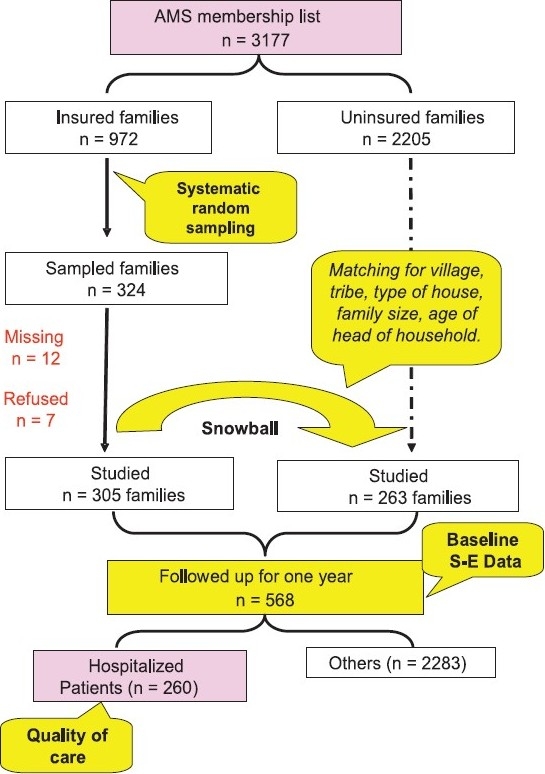
A schematic representation of the sampling method used at ACCORD, Gudalur. SE, socio-economic.

*Selection of study participants:* Household surveys were conducted both at ACCORD and KKVS to measure the satisfaction level of insured and uninsured patients. At ACCORD, a panel survey was conducted among both insured and uninsured households (Fig. 3). On July 1, 2004, there was a total of 972 (30%) insured and 2,205 uninsured households on the AMS membership list. A systematic random sample of 324 households was selected from the list of insured AMS members. A trained research team visited each of these 324 households to enrol them in the study. Of these, 12 had migrated and seven households refused to enrol (Fig. 3). Totally 305 insured households agreed to participate in the study. For each of the insured households enrolled, the team subsequently identified a matching uninsured household using a snowball technique. The households were matched on the basis of family size, age of the head of household, socio-economic status of the household, and distance from the ASHWINI hospital.

At KKVS, stratified random sampling was used to select the households. There were a total of 5,391 women who were members of the KKVS self-help groups in March 2005. Of these, 2,359 women and their families had enrolled in the KKVS CHI scheme for the period April 2004 - March 2005. Insured and uninsured members were then stratified according to geographic clusters and the proportion of insured and uninsured in each cluster was identified. Then 500 insured and 500 uninsured families were randomly sampled with probability proportional to the number of insured members in each cluster.

*Data collection:* Seven FGDs were conducted at ACCORD and three at KKVS to elicit the indicators for patient satisfaction as perceived by the respective communities. At ACCORD, a total of 37 men and 31 women participated in the FGDs while at KKVS, 29 women participated. The main questions asked were; (*i*) Where do you normally go for hospitalization? (*ii*) Why do you go there? and (*iii*) What do you understand by better care? The FGDs were taped and then subsequently transcribed and translated into English. Other than the community members, one FGD each was conducted among the field and nursing staff of these two organizations. Here the main objective was to understand their perception of why patients seek care in particular facilities.

**Fig. 4:**
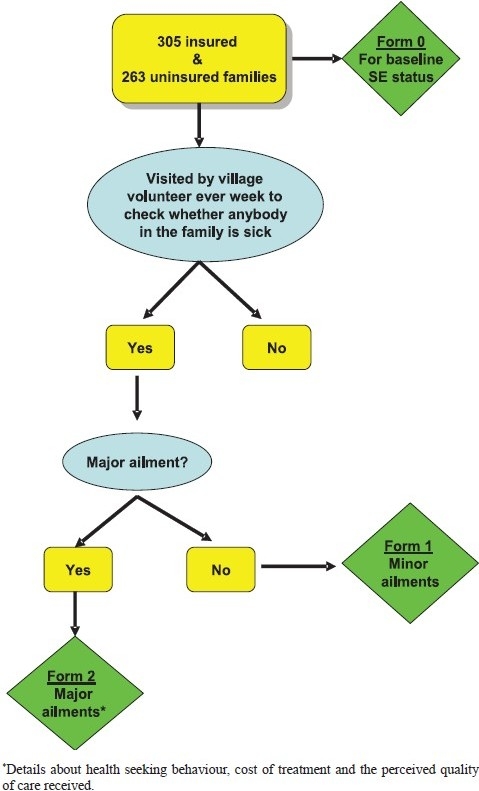
Interview schedules at ACCORD for the 568 households.

At ACCORD, all the sampled households were administered a structured baseline questionnaire (Form 0) by a trained interviewer at the beginning of the study to document the demographic and socioeconomic profile of the sampled households (Fig. 4). Each of these insured and uninsured households was visited on a weekly basis from July 1, 2004 to June 30, 2005 by village volunteers. During their visits, the volunteers recorded the presence or absence of any illness in the past week on a pre-printed questionnaire (Form 1). These questionnaires were handed over to a supervisor at the beginning of each month. The supervisor reviewed the submitted questionnaires and notified trained interviewers if there was a major ailment in any of the households. This interviewer then administered a third structured questionnaire (Form 2) to the patients who were hospitalized. The main elements investigated were: the utilization of hospital services; cost of treatment; and the satisfaction levels if admitted in a hospital. An insured member was defined as an AMS member who had paid the premium of 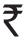 25 (US$ 0.54) for the period from July 2004 to June 2005.

At KKVS, each of these 1,000 members was approached by trained interviewers and administered a structured questionnaire. Their socio-economic profile, morbidity within the last one year, their health-seeking behaviour, the satisfaction levels when hospitalized and finally, the health expenditure on this event, were documented.

*Analysis:* The quantitative data were entered in MS Access and analysed, using SPSS for Windows version 10. 95% confidence intervals (CI) around the medians and proportions to indicate the precision of estimates were calculated. Non-parametric tests and χ^2^tests were used to assess whether the differences between the insured and the uninsured were statistically significant. The FGD data and the open-ended questions were analysed manually.

In both schemes, informed consent was obtained from the head of each households enrolled. Interviewees were assured that refusal to participate would have no consequence whatsoever. Confidentiality was maintained by including a unique identification number in the database for each interviewee. Ethical clearance was obtained from the Ethics board of SCTIMST – Thiruvananthapuram, India.

## Results

*ACCORD:* At ACCORD, a total of 305 insured and 263 uninsured households, with 1,444 and 1,225 individuals respectively, enrolled in the study. However, only 545 households had a baseline survey. These 545 households were followed regularly over 12 months. Matched parameters corresponded in both insured and uninsured households (Table II).

**Table II:** Characteristics of the sampled households at ACCORD and KKVS in 2004-2005

	ACCORD		KKVS	
	Insured	Uninsured	Insured	Uninsured
Number of households sampled	305	263	500	500
Number of households with baseline characteristics (individuals)	297 (1413)	248 (1173)	396 (1469)	412 (1517)
Median family size (95% CI)	5.0 (4.8, 5.2)	5.0 (4.8, 5.2)	4.0 (3.8,4.1)	4.0 (3.8, 4.2)
Median age of individuals (95% CI)	23.0 (21.9, 24.1)	22.0 (20.9, 23.1)	28 (27, 29)	25 (24, 26)
Proportion of females (95% CI)	52 (49, 54)	52 (49, 55)	53 (51, 56)	45 (43, 48)
Proportion of individuals (> 6 yr) who are literate (95% CI)	54 (51, 57)	52 (49, 55)	65 (63, 68)	77 (75, 80)
Median annual income/expenditure in Rs (95% CI)	28,520 (26,634, 30,452)	27,186 (25,714, 28,658)	28,198 (26,174, 30,222)	29,072 (26,634, 31,464)

A total of 183 insured and 77 uninsured individuals sought treatment at a formal health facility (Table III). The demographic, social and economic parameters of both groups of patients were similar.

**Table III:** Details of patients with major ailments who sought care with formal health services at ACCORD and KKVS in 2004-2005

	ACCORD		KKVS	
	Insured	Uninsured	Insured	Uninsured
Number of episodes of illness that were treated at a formal health facility (patients)	202 (183)	86 (77)	66 (66)	57 (57)
Median age in yr (95% CI)	24 (22, 25) *	25 (22, 30)	37 (35, 40)	31 (24, 45)
% of women (95% CI)	55 (48, 62)*	65 (54, 75)	50 (38, 62)	51 (38, 63)
% literate (95% CI)#	48 (40, 56)	50 (38, 62)	66 (54, 76)	73 (59, 83)+
Median household income/expenditure in Rs. (95% CI)	27,830 (24,702, 32,614)	25,806 (22,172, 27,692)	35,696 (32,430, 42,090)	34,040 (24,426, 38,456)

* Missing data

# Calculated on patients ≥ 6 yr

+ Missing data - 5

At ACCORD, 82 per cent of the insured patients were generally satisfied with the care received, the corresponding figure for uninsured patients was 73 per cent (Table IV). While the insured had a higher level of satisfaction, this difference was not significant. Satisfaction was similar across socioeconomic and demographic variables. It appears that age, gender, literacy and economic status did not determine satisfaction levels. The reasons for satisfaction were similar in both insured and uninsured. Both insured and uninsured were happy with the infrastructure (84 and 78% respectively). The service orientation of the doctors and nurses were slightly less satisfactory, but the difference between the insured and uninsured patients was not significant. However, only half the patients (both insured and uninsured) were content with the processes by which they received care. Almost all insured and uninsured felt better at the end of the treatment (Table IV). The only advantage that the insured had was a shorter waiting time compared to the uninsured patients (*P*<0.05).

**Table IV:** Proportion of patients (%) who were satisfied with care received, at ACCORD and KKVS and the reasons therein (95 CI)

Measures	ACCORD		KKVS	
	Insured (n= 202)	Uninsured (n = 86)	Insured (n = 66)	Uninsured (n = 57)
I was satisfied with the care received	92 (88, 95)	87 (89, 94)	95 (90, 100)*	79 (68, 89)#
The medicines were effective	89 (84, 93)	85 (77, 92)	100 (94, 100)α	91 (83, 98)*
I felt cared for	88 (84, 92)	81 (73, 90)	98 (95, 101)α	89 (80, 97)α
I felt better	98 (96, 100)α	98(94,101)	94 (88, 100)	93 (86. 100)#
*Overall satisfaction: proportion of all positive responses*	82 (76, 87)	73 (64, 83)	89 (81, 97)	80 (69, 91)
I have faith in the doctor	88 (83, 92)	85 (77, 92)	98 (95, 101)α	91 (83, 98)*
Doctor explained about my illness	77 (71, 83)	73 (64, 83)	98 (95, 101)ε	94 (88, 100)α
I received instructions at discharge	80 (75, 86)	78 (69, 87)	97 (92, 101)ε	96 (91, 101)*
Doctor listened to my problems	84 (79, 89)	79 (70, 88)	98 (95, 101)α	100 (94, 100)*
Doctor examined me	88 (83, 92)	85 (77, 92)	100 (94, 100)α	98 (95, 102)*
*Doctors’ service orientation: proportion of all positive responses*	65 (58, 71)	60 (50, 70)	91 (83, 98)	85 (76, 95)
Staff did not shout at me	96 (93, 98)	95 (91, 100)	84 (75, 93)α	69 (57, 81)*
I was received warmly	88 (84, 92)	90 (83, 96)	98 (95, 101)α	95 (89, 101)*
Staff were courteous	86 (81, 90)	81 (73, 90)	97 (92, 101)α	96 (91, 101)*
I was not afraid	91 (87, 95)	92 (86, 98)	73 (62, 84)α	65 (53, 78)*
*Nurses’ service orientation: proportion of all positive responses*	73 (67, 79)	74 (65, 84)	63 (51, 75)	44 (31, 57)
I was satisfied with the amenities	86 (81, 90)	78 (69, 87)	94 (88, 100)α	100 (94, 100)*
I received medicines at the hospital	88 (83, 92)	87 (80, 94)	94 (87, 100)ε	98 (95, 102)*
*Tangibles: proporion of all positive responses*	84 (79, 89)	78 (69, 87)	86 (77, 94)	98 (95, 102)
Waiting period was not long	94 (90, 97)β	85 (77, 93)δ	78 (68, 88)α	60 (47, 74)ε
The treatment was not costly	87 (82, 92)	92 (86, 98)	32 (21, 44)ε	20 (10, 31)α
Visitors were allowed to see me	68 (62, 75)	62 (51, 72)	87 (79, 95)ε	89 (81, 97)*
I did not pay informal fees	98 (96, 100)	97 (93, 100)	87 (79, 95)ε	72 (60, 84)α
*Processes: proporion of all positive responses*	53 (46, 60)	48 (37, 58)	16 (7, 25)	9 (2, 17)

# 1 non responder

* 2 non responders

± 3 non responders

² 19 non responders

ε 4 non responder

δ 9 non responders

Confidence interval calculated from the following website: http://www.dimensionresearch.com/resources/calculators/conf_prop.html

The open-ended questions clearly showed that, for both the insured and uninsured adivasis in Gudalur, the main reasons for satisfaction were that they “received good treatment / good medicines” (32% of responses) and “felt better / healed / cured” (28% of responses by insured and 24% of responses by uninsured).

The main reason for dissatisfaction was the poor outcome of the therapy. Usually the patient had expired or continued to have symptoms in spite of treatment. Of all the patients at ACCORD, 80 per cent of insured and 66 per cent of uninsured went to the NGO facility. Only 12 and 24 per cent of insured and uninsured respectively used the private facility. Those who went to a private provider were less likely to receive medicines, to be treated courteously by the staff, to have satisfactory facilities, to have visitors call on them, to feel cared for or to receive affordable treatment.

Focus group discussion with the staff at ASHWINI indicated that they did not differentiate between the insured and uninsured. While they were aware of the insurance status, all patients received similar treatment. However, some uninsured people disagreed, stating that sometimes, the nurses in the hospital would reproach them for ‘being uninsured’. Hence they were uncomfortable coming to the NGO Hospital. Some of the staff also considered the insured patients as a nuisance.

### 

#### KKVS

At KKVS, while a total of 1,000 families were sampled, only 808 were available. The rest had migrated to urban areas at the time of the survey mostly for employment purposes. Three hundred and ninety six of the insured families and 412 of the uninsured families enrolled in the study. The median family size in both insured and uninsured categories was similar and both insured and uninsured families belonged to similar economic strata (Table II). However, the insured individuals tend to be older and less literate compared to the uninsured. There were more women in the insured families as compared to the uninsured.

Sixty six insured and 57 uninsured patients sought care with formal health services. While both insured and uninsured patients had similar socio-economic status, it is important to note that in both categories the older, more literate and wealthier people used the health services (Table III).

At KKVS, 95 per cent of insured and only 79 per cent of uninsured patients were satisfied with the care received (Table IV). This difference was statistically significant (*P*<0.01).

One of the reasons for satisfaction among the insured was that the staff did not shout at them (*P*<0.05). Also the insured were seen faster compared to the uninsured patients (*P*<0.05). And finally, less number of insured patients had to pay informal fees compared to the uninsured (*P*<0.05). While more uninsured found the treatment costly, the medicines less effective and did not have faith in the doctor; the differences were not very significant.

Unlike at ACCORD, most of the patients at DHAN-KKVS used either the private or public facility. Sixty two per cent of the insured used the private sector, while the corresponding figure for the uninsured was 44 per cent (*P*<0.01). Forty nine per cent of the uninsured also used the government facility. Patients used the private sector probably due to two reasons, one was the reduction in the financial barrier and the other was the perception that the private sector provided better quality of care. This should be translated into higher satisfaction levels by patients using the private sector. However, 92 per cent of those patients who used the private sector were satisfied, 84 per cent of the patients who used the government sector were also satisfied.

When the above three reasons were disaggregated for increased satisfaction by the type of provider, it was found that in the private sector, the insured had a lower probability of being shouted at (*P*<0.01). However, this relationship was not seen for either the waiting period or informal fees.

At KKVS, 50 per cent of the insured patients had visited more than two health facilities before getting cured, while among the uninsured, the figure was 44 per cent. However, more uninsured preferred to use the tertiary level compared to the insured [32% (95% CI: 20, 45) and 9% (95% CI: 3, 19) respectively].

DHAN empanelled the providers based on their capacity to provide medical and surgical care and further negotiated with the hospitals to reduce the fees for insured patients; and to provide the documents to the patients as soon as possible. Patients felt that the doctors charged higher fees for insured patients. This affected the patient directly, as the patient had to pay 25 per cent of the total bill. Hence the patients usually hid their insurance status till the time of the discharge.

## Discussion

Our study shows that while both at ACCORD and DHAN-KKVS the insured patients had higher satisfaction levels compared to the uninsured patients, this difference was not statistically significant at ACCORD. The main reason for satisfaction was the outcome of the treatment. Patients who were cured or healed had a higher probability of being satisfied.

The indicators used for measuring patient satisfaction were drawn from literature and finetuned through focus group discussions with patients. However, we could not validate this by independently observing whether the patient actually received the quality that they perceived. This is a drawback in this study. Yet another limitation is that we used a dichotomous scale for measuring the satisfaction levels. We may have received a more qualified response by using a wider scale. The insured are usually risk averse and hence enrol into the insurance programme. This self selection may have some influence on their perceptions of satisfaction. Unfortunately, we could not measure this and its effect on the results. This would probably be negligible, given the fact that there was no significant difference in the observable determinants of utilization between insured and uninsured. Finally, the insured may have had more interactions with the health services, and hence, less expectations. Thus their threshold of satisfaction would have been less, compared to the uninsured. However, as we did not measure the expectations *a priori*, it could not be commented upon.

Interviews with 383 hospitalized patients at two different locations, more than 1,000 km apart; showed that there was very little difference in the satisfaction levels of insured and uninsured patients. Our hypothesis was that the insurance scheme would have negotiated for better quality of care for its members and so the insured would have received better quality of care and thereby would be more satisfied. Another assumption was that the insured patients would be more aware of their rights and would have demanded for better services. However, our findings showed that the satisfaction levels among both insured and uninsured patients were similar especially in ACCORD.

A major reason could be the large social gap between the provider and the patient. This would have prevented the patient from ‘demanding’ better quality of care. More than 10 per cent of insured in both ACCORD and KKVS had to wait for a long time, were not examined by the doctor, or did not receive medicines. Insured patients were labelled by some staff as “nuisance” and “free loaders” who would demand ’unnecessary services’. Further, there was very little or no strategic purchasing of healthcare by the insurers on behalf of the insured in both the CHI schemes. At DHAN-KKVS, the insurance co-ordinator negotiated for lower fees and for appropriate documentation. But there was no effort to use the leverage of pooled funds to obtain certain privileges for the insured patients. In neither of the schemes was there any explicit negotiation process or formal documents to indicate that strategic purchasing had taken place. This is probably due to the fact that neither KKVS nor AMS had the technical capacity to parley with the providers. This is a major weakness of most CHI schemes, not just in India but also internationally^24^. Also it was not explicitly part of the objectives of the two schemes^25^. So, a third party needs to step in to ensure adequate services for the insured patients. One possible solution is to have a technically competent third party negotiate on behalf of the community. This could be a government representative, or a not-for-profit but competent body.

A third reason could be that the healthcare providers offered similar good quality care to patients, irrespective of their insurance status. Thus, from a system perspective, one could say that the CHI scheme has been effective in improving the quality of care for all. While in terms of equity, this may seem reasonable, for the members of the insurance scheme it may be a disincentive. They may not perceive any difference between themselves and their uninsured neighbour while seeking care.

Our study showed a high level of satisfaction regarding the care received both among the insured and uninsured patients. This is surprising as reportedly the quality of care in the Indian health services is low. Peter Berman in 1998 stated clearly that the quality of care in both public and primary health services was low^26^. In a community-based study, Pai^11^found that 45 per cent of women who had delivered in Madras had undergone a Caesarean section. Ramanathan *et al*^27^. observed that government doctors conducted 48 laparoscopic surgeries in two hours, did not counsel the patients and neglected aseptic measures. Peters *et al*^28^had commented on the lack of standards to ensure quality in India. Despite such empirical evidence of poor quality, patients were satisfied with the care received. This could be because of low expectations. Indeed perception of quality is dependent on various factors, an important one being the expectations of the patient^29^. This could explain the high levels of satisfaction, though technically they may have received poor quality care.

The main reasons for dissatisfaction were associated with poor outcomes of treatment, indicating that this was one of the valued expectations from a consultation. The insured patients at KKVS tended to use the private sector more than the uninsured. This is probably due to a combination of reduced financial barriers and a perception that private sector provides better care. However, the levels of dissatisfaction were higher among those patients who visited the private sector, both at ACCORD and KKVS.

One of the reasons for choosing two different CHI schemes was to see whether the design of the scheme had any effect on satisfaction levels. It was noted that patients at KKVS were more satisfied with the care received in four of the five dimensions as compared to those at ACCORD. Could this be due to the fact that the patients at KKVS had more choice of providers? On the other hand, patients at ACCORD were limited to only one provider and may have felt restricted.

Meeting patients’ expectations is an important step towards providing continuous high quality healthcare^30^. It has the potential to make patients adhere to the care provided and return for follow up^20^. This is more important in a CHI scheme, where dissatisfied patients may refuse to renew their membership in the next year^31^,^32^. They may dissuade others from joining the scheme; thereby affecting the overall viability of the scheme. Hence it is imperative that CHI scheme managers ensure that the insured receive a high quality of care and are satisfied with the services. This, along with other measures, like affordable premium, acceptable benefit package, easy administrative procedures and trust in the organization would go a long way in ensuring the success of CHI schemes^33^. In India, there is a clear need for the poor to receive good quality health care and the CHI schemes should ensure that the objective is achieved.
